# Supplementary material for: Overlapping cell population expression profiling and regulatory inference in C. elegans
Source: BMC Genomics. 2016 Feb 29;17:159. doi: 10.1186/s12864-016-2482-z (PMC4772325; doi:10.1186/s12864-016-2482-z)
Supplement: Additional file 13: — Web supplement. (DOC 21 kb) [file 12864_2016_2482_MOESM13_ESM.zip › sortWeb/clusters/hier.300.clusters/73.html]

Cluster 73 

## Cluster 73

### Expression

| cnd-1 rep. 1 | cnd-1 rep. 2 | cnd-1 rep. 3 | pha-4 rep. 1 | pha-4 rep. 2 | pha-4 rep. 3 | ceh-27 | ceh-36 | ceh-6 | F21D5.9 | mir-57 | mls-2 | pal-1 | pros-1 | ttx-3 | unc-130 | hlh-16 | irx-1 | ceh-6 (+) hlh-16 (+) | ceh-6 (+) hlh-16 (-) | ceh-6 (-) hlh-16 (+) | cnd-1 singlets | pha-4 singlets | 0 | 60 | 120 | 150 | 180 | 240 | 330 | 390 | 420 | 480 | 540 | 570 | 600 | 630 | 660 | NAME | Functional description |
| --- | --- | --- | --- | --- | --- | --- | --- | --- | --- | --- | --- | --- | --- | --- | --- | --- | --- | --- | --- | --- | --- | --- | --- | --- | --- | --- | --- | --- | --- | --- | --- | --- | --- | --- | --- | --- | --- | --- | --- |
|  |  |  |  |  |  |  |  |  |  |  |  |  |  |  |  |  |  |  |  |  |  |  |  |  |  |  |  |  |  |  |  |  |  |  |  |  |  | *mtl-1* | MeTaLlothionein |
|  |  |  |  |  |  |  |  |  |  |  |  |  |  |  |  |  |  |  |  |  |  |  |  |  |  |  |  |  |  |  |  |  |  |  |  |  |  | *pcp-4* | Prolyl Carboxy Peptidase like |
|  |  |  |  |  |  |  |  |  |  |  |  |  |  |  |  |  |  |  |  |  |  |  |  |  |  |  |  |  |  |  |  |  |  |  |  |  |  | K09C8.7 |  |
|  |  |  |  |  |  |  |  |  |  |  |  |  |  |  |  |  |  |  |  |  |  |  |  |  |  |  |  |  |  |  |  |  |  |  |  |  |  | T05F1.9 |  |
|  |  |  |  |  |  |  |  |  |  |  |  |  |  |  |  |  |  |  |  |  |  |  |  |  |  |  |  |  |  |  |  |  |  |  |  |  |  | C16B8.3 |  |
|  |  |  |  |  |  |  |  |  |  |  |  |  |  |  |  |  |  |  |  |  |  |  |  |  |  |  |  |  |  |  |  |  |  |  |  |  |  | *hrg-6* | Heme Responsive Gene |
|  |  |  |  |  |  |  |  |  |  |  |  |  |  |  |  |  |  |  |  |  |  |  |  |  |  |  |  |  |  |  |  |  |  |  |  |  |  | Y110A7A.21 |  |
|  |  |  |  |  |  |  |  |  |  |  |  |  |  |  |  |  |  |  |  |  |  |  |  |  |  |  |  |  |  |  |  |  |  |  |  |  |  | R09H10.3 |  |
|  |  |  |  |  |  |  |  |  |  |  |  |  |  |  |  |  |  |  |  |  |  |  |  |  |  |  |  |  |  |  |  |  |  |  |  |  |  | F25D1.8 |  |
|  |  |  |  |  |  |  |  |  |  |  |  |  |  |  |  |  |  |  |  |  |  |  |  |  |  |  |  |  |  |  |  |  |  |  |  |  |  | B0198.4 |  |
|  |  |  |  |  |  |  |  |  |  |  |  |  |  |  |  |  |  |  |  |  |  |  |  |  |  |  |  |  |  |  |  |  |  |  |  |  |  | *cnp-3* | CalciNeurin binding Protein |
|  |  |  |  |  |  |  |  |  |  |  |  |  |  |  |  |  |  |  |  |  |  |  |  |  |  |  |  |  |  |  |  |  |  |  |  |  |  | C23F12.7 |  |
|  |  |  |  |  |  |  |  |  |  |  |  |  |  |  |  |  |  |  |  |  |  |  |  |  |  |  |  |  |  |  |  |  |  |  |  |  |  | T22C8.4 |  |
|  |  |  |  |  |  |  |  |  |  |  |  |  |  |  |  |  |  |  |  |  |  |  |  |  |  |  |  |  |  |  |  |  |  |  |  |  |  | C18C4.4 |  |
|  |  |  |  |  |  |  |  |  |  |  |  |  |  |  |  |  |  |  |  |  |  |  |  |  |  |  |  |  |  |  |  |  |  |  |  |  |  | *nspd-1* | Nematode Specific Peptide family, group D |
|  |  |  |  |  |  |  |  |  |  |  |  |  |  |  |  |  |  |  |  |  |  |  |  |  |  |  |  |  |  |  |  |  |  |  |  |  |  | ZK783.8 |  |
|  |  |  |  |  |  |  |  |  |  |  |  |  |  |  |  |  |  |  |  |  |  |  |  |  |  |  |  |  |  |  |  |  |  |  |  |  |  | C41D7.1 |  |
|  |  |  |  |  |  |  |  |  |  |  |  |  |  |  |  |  |  |  |  |  |  |  |  |  |  |  |  |  |  |  |  |  |  |  |  |  |  | T22A3.11 |  |
|  |  |  |  |  |  |  |  |  |  |  |  |  |  |  |  |  |  |  |  |  |  |  |  |  |  |  |  |  |  |  |  |  |  |  |  |  |  | *srbc-27* | Serpentine Receptor, class BC (class B-like) |
|  |  |  |  |  |  |  |  |  |  |  |  |  |  |  |  |  |  |  |  |  |  |  |  |  |  |  |  |  |  |  |  |  |  |  |  |  |  | ZC373.8 |  |
|  |  |  |  |  |  |  |  |  |  |  |  |  |  |  |  |  |  |  |  |  |  |  |  |  |  |  |  |  |  |  |  |  |  |  |  |  |  | F57B10.16 |  |
|  |  |  |  |  |  |  |  |  |  |  |  |  |  |  |  |  |  |  |  |  |  |  |  |  |  |  |  |  |  |  |  |  |  |  |  |  |  | *srx-120* | Serpentine Receptor, class X |
|  |  |  |  |  |  |  |  |  |  |  |  |  |  |  |  |  |  |  |  |  |  |  |  |  |  |  |  |  |  |  |  |  |  |  |  |  |  | T22D1.20 |  |
|  |  |  |  |  |  |  |  |  |  |  |  |  |  |  |  |  |  |  |  |  |  |  |  |  |  |  |  |  |  |  |  |  |  |  |  |  |  | *lys-6* | LYSozyme |
|  |  |  |  |  |  |  |  |  |  |  |  |  |  |  |  |  |  |  |  |  |  |  |  |  |  |  |  |  |  |  |  |  |  |  |  |  |  | C01B7.10 |  |
|  |  |  |  |  |  |  |  |  |  |  |  |  |  |  |  |  |  |  |  |  |  |  |  |  |  |  |  |  |  |  |  |  |  |  |  |  |  | F07C3.14 |  |
|  |  |  |  |  |  |  |  |  |  |  |  |  |  |  |  |  |  |  |  |  |  |  |  |  |  |  |  |  |  |  |  |  |  |  |  |  |  | T03G11.14 |  |
|  |  |  |  |  |  |  |  |  |  |  |  |  |  |  |  |  |  |  |  |  |  |  |  |  |  |  |  |  |  |  |  |  |  |  |  |  |  | T02E9.19 |  |
|  |  |  |  |  |  |  |  |  |  |  |  |  |  |  |  |  |  |  |  |  |  |  |  |  |  |  |  |  |  |  |  |  |  |  |  |  |  | *ubc-23* | UBiquitin Conjugating enzyme |
|  |  |  |  |  |  |  |  |  |  |  |  |  |  |  |  |  |  |  |  |  |  |  |  |  |  |  |  |  |  |  |  |  |  |  |  |  |  | C26D10.3 |  |
|  |  |  |  |  |  |  |  |  |  |  |  |  |  |  |  |  |  |  |  |  |  |  |  |  |  |  |  |  |  |  |  |  |  |  |  |  |  | *dct-3* | DAF-16/FOXO Controlled, germline Tumor affecting |
|  |  |  |  |  |  |  |  |  |  |  |  |  |  |  |  |  |  |  |  |  |  |  |  |  |  |  |  |  |  |  |  |  |  |  |  |  |  | H32K16.1 |  |
|  |  |  |  |  |  |  |  |  |  |  |  |  |  |  |  |  |  |  |  |  |  |  |  |  |  |  |  |  |  |  |  |  |  |  |  |  |  | *ulp-4* | Ubiquitin-Like Protease |
|  |  |  |  |  |  |  |  |  |  |  |  |  |  |  |  |  |  |  |  |  |  |  |  |  |  |  |  |  |  |  |  |  |  |  |  |  |  | T23E1.6 |  |
|  |  |  |  |  |  |  |  |  |  |  |  |  |  |  |  |  |  |  |  |  |  |  |  |  |  |  |  |  |  |  |  |  |  |  |  |  |  | ZK177.1 |  |
|  |  |  |  |  |  |  |  |  |  |  |  |  |  |  |  |  |  |  |  |  |  |  |  |  |  |  |  |  |  |  |  |  |  |  |  |  |  | C31B8.4 |  |
|  |  |  |  |  |  |  |  |  |  |  |  |  |  |  |  |  |  |  |  |  |  |  |  |  |  |  |  |  |  |  |  |  |  |  |  |  |  | F15D3.9 |  |
|  |  |  |  |  |  |  |  |  |  |  |  |  |  |  |  |  |  |  |  |  |  |  |  |  |  |  |  |  |  |  |  |  |  |  |  |  |  | F14F9.8 |  |
|  |  |  |  |  |  |  |  |  |  |  |  |  |  |  |  |  |  |  |  |  |  |  |  |  |  |  |  |  |  |  |  |  |  |  |  |  |  | K09C8.2 |  |
|  |  |  |  |  |  |  |  |  |  |  |  |  |  |  |  |  |  |  |  |  |  |  |  |  |  |  |  |  |  |  |  |  |  |  |  |  |  | *fbxb-99* | F-box B protein |
|  |  |  |  |  |  |  |  |  |  |  |  |  |  |  |  |  |  |  |  |  |  |  |  |  |  |  |  |  |  |  |  |  |  |  |  |  |  | T16G12.4 |  |
|  |  |  |  |  |  |  |  |  |  |  |  |  |  |  |  |  |  |  |  |  |  |  |  |  |  |  |  |  |  |  |  |  |  |  |  |  |  | *mom-2* | More Of MS |
|  |  |  |  |  |  |  |  |  |  |  |  |  |  |  |  |  |  |  |  |  |  |  |  |  |  |  |  |  |  |  |  |  |  |  |  |  |  | *psa-3* | Phasmid Socket Absent |
|  |  |  |  |  |  |  |  |  |  |  |  |  |  |  |  |  |  |  |  |  |  |  |  |  |  |  |  |  |  |  |  |  |  |  |  |  |  | *php-3* | Posterior Hox gene Paralog |
|  |  |  |  |  |  |  |  |  |  |  |  |  |  |  |  |  |  |  |  |  |  |  |  |  |  |  |  |  |  |  |  |  |  |  |  |  |  | *nob-1* | kNOB-like posterior (NO Backside) |
|  |  |  |  |  |  |  |  |  |  |  |  |  |  |  |  |  |  |  |  |  |  |  |  |  |  |  |  |  |  |  |  |  |  |  |  |  |  | Y47D3B.11 |  |
|  |  |  |  |  |  |  |  |  |  |  |  |  |  |  |  |  |  |  |  |  |  |  |  |  |  |  |  |  |  |  |  |  |  |  |  |  |  | *mig-14* | abnormal cell MIGration |
|  |  |  |  |  |  |  |  |  |  |  |  |  |  |  |  |  |  |  |  |  |  |  |  |  |  |  |  |  |  |  |  |  |  |  |  |  |  | *pal-1* | Posterior ALae in males |
|  |  |  |  |  |  |  |  |  |  |  |  |  |  |  |  |  |  |  |  |  |  |  |  |  |  |  |  |  |  |  |  |  |  |  |  |  |  | K10B4.1 |  |
|  |  |  |  |  |  |  |  |  |  |  |  |  |  |  |  |  |  |  |  |  |  |  |  |  |  |  |  |  |  |  |  |  |  |  |  |  |  | *bath-15* | BTB and MATH domain containing |
|  |  |  |  |  |  |  |  |  |  |  |  |  |  |  |  |  |  |  |  |  |  |  |  |  |  |  |  |  |  |  |  |  |  |  |  |  |  | *tlp-1* | T lineage defect, LePtoderan tail |
|  |  |  |  |  |  |  |  |  |  |  |  |  |  |  |  |  |  |  |  |  |  |  |  |  |  |  |  |  |  |  |  |  |  |  |  |  |  | *cwn-1* | C. elegans WNT family |
|  |  |  |  |  |  |  |  |  |  |  |  |  |  |  |  |  |  |  |  |  |  |  |  |  |  |  |  |  |  |  |  |  |  |  |  |  |  | *mab-5* | Male ABnormal |
|  |  |  |  |  |  |  |  |  |  |  |  |  |  |  |  |  |  |  |  |  |  |  |  |  |  |  |  |  |  |  |  |  |  |  |  |  |  | C46C11.5 |  |
|  |  |  |  |  |  |  |  |  |  |  |  |  |  |  |  |  |  |  |  |  |  |  |  |  |  |  |  |  |  |  |  |  |  |  |  |  |  | R11H6.7 |  |
|  |  |  |  |  |  |  |  |  |  |  |  |  |  |  |  |  |  |  |  |  |  |  |  |  |  |  |  |  |  |  |  |  |  |  |  |  |  | F52B10.14 |  |
|  |  |  |  |  |  |  |  |  |  |  |  |  |  |  |  |  |  |  |  |  |  |  |  |  |  |  |  |  |  |  |  |  |  |  |  |  |  | *linc-116* | Long Intervening Non-Coding RNA |
|  |  |  |  |  |  |  |  |  |  |  |  |  |  |  |  |  |  |  |  |  |  |  |  |  |  |  |  |  |  |  |  |  |  |  |  |  |  | C26D10.7 |  |
|  |  |  |  |  |  |  |  |  |  |  |  |  |  |  |  |  |  |  |  |  |  |  |  |  |  |  |  |  |  |  |  |  |  |  |  |  |  | W04E12.7 |  |
|  |  |  |  |  |  |  |  |  |  |  |  |  |  |  |  |  |  |  |  |  |  |  |  |  |  |  |  |  |  |  |  |  |  |  |  |  |  | Y75B8A.50 |  |
|  |  |  |  |  |  |  |  |  |  |  |  |  |  |  |  |  |  |  |  |  |  |  |  |  |  |  |  |  |  |  |  |  |  |  |  |  |  | W06D12.6 |  |
|  |  |  |  |  |  |  |  |  |  |  |  |  |  |  |  |  |  |  |  |  |  |  |  |  |  |  |  |  |  |  |  |  |  |  |  |  |  | Y95B8A.14 |  |
|  |  |  |  |  |  |  |  |  |  |  |  |  |  |  |  |  |  |  |  |  |  |  |  |  |  |  |  |  |  |  |  |  |  |  |  |  |  | *fbxa-218* | F-box A protein |

### Phenotypes enriched

|  |  |  |  |
| --- | --- | --- | --- |
| **Group name** | **Number in cluster** | **Enrichment** | **FDR corrected p** |
| male tail sensory ray development variant | 4 | 27.66 | 0.016 |
| male nervous system development variant | 4 | 27.66 | 0.016 |

### Anatomy terms enriched

none found

### GO terms enriched

|  |  |  |
| --- | --- | --- |
| **GO term** | **Number of genes** | **FDR-corrected p-value** |
| Wnt signaling pathway | 3 | 0.011 |

### Expression clusters enriched

none found

### Motifs enriched

|  |  |  |  |  |  |
| --- | --- | --- | --- | --- | --- |
| **Motif** | **Logo** | **Possible orthologs** | **Number of motifs in cluster** | **Enrichment** | **FDR corrected p** |
| pTH3467 |  | nhr-6 | 31 | 2.04 | 0.0022 |
| HOXA10\_2 |  | pal-1 (0.93) php-3 (0.91) lin-39 (0.67) ceh-24 | 31 | 1.94 | 0.0051 |
| Hth\_SOLEXA\_FBgn0001235 |  | ceh-32 | 29 | 2.01 | 0.0057 |
| pTH9135 |  | pop-1 | 27 | 2.07 | 0.0069 |
| HXA9\_f1 |  | lin-39 (0.67) | 14 | 3.23 | 0.0087 |
| T-47D\_GATA3\_HudsonAlpha |  | elt-1 (0.65) | 8 | 5.63 | 0.0091 |
| ELF3\_f1 |  | C24A1.2 | 29 | 1.94 | 0.0092 |
| Hoxa13\_3126 |  | pal-1 (0.93) ceh-24 | 29 | 1.93 | 0.0100 |
| Egr3\_1 |  | ZC328.2 | 32 | 1.80 | 0.0120 |
| Hoxd13\_2356 |  | pal-1 (0.93) | 30 | 1.87 | 0.0130 |
| Aef1\_FlyReg\_FBgn0005694 |  | K11D2.4 | 62 | 1.18 | 0.0150 |
| MA0070.1 |  | ceh-20 (0.7) | 59 | 1.24 | 0.0160 |
| pTH9214 |  | cfi-1 | 33 | 1.74 | 0.0160 |
| pTH5267 |  | hlh-32 (0.59) | 30 | 1.83 | 0.0160 |
| MA0296.1 |  | lin-31 fkh-8 | 29 | 1.87 | 0.0170 |
| V$TST1\_01 |  | pal-1 (0.93) ceh-18 | 25 | 2.02 | 0.0180 |
| MEF2A\_f1 |  | mef-2 | 12 | 3.34 | 0.0180 |
| HOXC10\_1 |  | php-3 (0.91) lin-39 (0.67) hbl-1 | 44 | 1.49 | 0.0180 |
| pTH5539 |  | unc-120 (0.69) | 57 | 1.27 | 0.0180 |
| pTH9246 |  | C34D1.1 | 18 | 2.45 | 0.0190 |
| V$FREAC4\_01 |  | let-381 | 51 | 1.36 | 0.0200 |
| pTH10795 |  | ztf-14 | 49 | 1.39 | 0.0210 |
| POU3F4\_2 |  | ceh-18 | 14 | 2.88 | 0.0210 |
| NEUROD2\_1 |  | hlh-8 (0.69) ngn-1 | 22 | 2.11 | 0.0250 |
| V$GATA1\_05 |  | elt-1 (0.65) | 14 | 2.82 | 0.0250 |
| ETV4\_f1 |  | lin-1 | 42 | 1.49 | 0.0270 |
| Msx3\_1 |  | ceh-1 | 63 | 1.13 | 0.0310 |
| ARI3A\_do |  | cfi-1 | 27 | 1.83 | 0.0360 |
| pTH9052 |  | ces-2 (0.58) | 58 | 1.23 | 0.0370 |
| POU3F1\_1 |  | ceh-18 | 29 | 1.75 | 0.0380 |
| pTH9298 |  | attf-1 | 46 | 1.40 | 0.0400 |
| Mv73 |  | elt-1 (0.65) | 48 | 1.37 | 0.0400 |
| STF1\_f1 |  | nhr-68 | 50 | 1.34 | 0.0400 |
| ELF1\_f1 |  | lin-1 C24A1.2 | 43 | 1.44 | 0.0410 |
| pTH5164 |  | hlh-32 (0.59) | 17 | 2.33 | 0.0410 |
| Cdx1\_2245 |  | ceh-13 | 10 | 3.40 | 0.0420 |
| pTH5098 |  | F45H11.6 | 38 | 1.53 | 0.0420 |
| I$E74A\_01 |  | C24A1.2 | 49 | 1.35 | 0.0430 |
| pTH5159 |  | aha-1 | 10 | 3.36 | 0.0440 |
| V$TATA\_C |  | tbp-1 | 26 | 1.83 | 0.0450 |
| FOXJ3\_f2 |  | lin-31 | 19 | 2.16 | 0.0450 |
| MA0531.1 |  | Y5F2A.4 | 34 | 1.60 | 0.0470 |
| pTH9296 |  | C34D1.1 | 24 | 1.90 | 0.0470 |
| Dfd\_SOLEXA\_FBgn0000439 |  | lin-39 (0.67) | 18 | 2.21 | 0.0480 |
| MA0544.1 |  | gei-11 | 16 | 2.37 | 0.0490 |

### Correlated (and anti-correlated) transcription factors

|  |  |
| --- | --- |
| **Transcription factor** | **Correlation** |
| tlp-1 | 0.94 |
| pal-1 | 0.93 |
| nob-1 | 0.92 |
| php-3 | 0.91 |
| mab-5 | 0.83 |
| T22C8.4 | 0.81 |
| egl-5 | 0.80 |
| K05F1.5 | 0.79 |
| ceh-99 | 0.78 |
| cog-1 | 0.77 |
| nhr-111 | 0.76 |
| nhr-9 | 0.75 |
| nhr-69 | 0.73 |
| elt-3 | 0.72 |
| ehn-3 | 0.72 |
| dmd-3 | 0.72 |
| C38D4.7 | 0.70 |
| ceh-20 | 0.70 |
| mls-1 | 0.70 |
| sea-1 | 0.69 |
| unc-120 | 0.69 |
| hlh-8 | 0.69 |
| tbx-41 | 0.67 |
| lin-39 | 0.67 |
| pat-9 | 0.66 |
| ceh-62 | -0.53 |
| nhr-198 | -0.53 |
| nhr-268 | -0.53 |
| nhr-51 | -0.54 |
| nhr-159 | -0.54 |
| sox-4 | -0.54 |
| nhr-167 | -0.54 |
| K12H6.12 | -0.54 |
| nhr-197 | -0.54 |
| nhr-52 | -0.54 |
| zfh-2 | -0.56 |
| nhr-21 | -0.56 |
| nhr-50 | -0.56 |
| lfi-1 | -0.56 |
| sox-3 | -0.57 |
| F21G4.5 | -0.59 |
| nhr-222 | -0.59 |
| C06E2.1 | -0.59 |
| nhr-71 | -0.60 |
| sox-2 | -0.60 |
| fkh-2 | -0.60 |
| nhr-286 | -0.60 |
| nhr-124 | -0.60 |
| nhr-275 | -0.69 |
| nhr-215 | -0.73 |

### ChIP peaks enriched

|  |  |  |  |  |
| --- | --- | --- | --- | --- |
| **Gene** | **Experiment** | **Number of upstream peaks** | **Enrichment** | **FDR corrected p** |
| mab-5 | MAB-5\_Embryos | 7 | 11.78 | 0.00013 |
| egl-5 | EGL-5\_Larvae-L3-stage | 13 | 2.63 | 0.03400 |
